# Evaluation of Antioxidant, Immunomodulatory, and Cytotoxic Action of Fractions from *Eugenia uniflora* L. and *Eugenia malaccensis* L.: Correlation with Polyphenol and Flavanoid Content

**DOI:** 10.1155/2013/125027

**Published:** 2013-09-05

**Authors:** Evellyne de Oliveira Figueirôa, Luís Cláudio Nascimento da Silva, Cristiane Moutinho Lagos de Melo, Juliana Kelle de Andrade Lemoine Neves, Nicácio Henrique da Silva, Valéria Rêgo Alves Pereira, Maria Tereza dos Santos Correia

**Affiliations:** ^1^Laboratório de Glicoproteínas, Departamento de Bioquímica, Centro de Ciências Biológicas, Universidade Federal de Pernambuco, Avenida Professor Moraes Rêgo s/n, Cidade Universitária, 50670-420 Recife, PE, Brazil; ^2^Laboratório de Imunoparasitologia, Departamento de Imunologia, Centro de Pesquisas Aggeu Magalhães, Fundação Oswaldo Cruz/PE, Avenida Professor Moraes Rego s/n, Cidade Universitária, 50670-420 Recife, PE, Brazil; ^3^Laboratório de Produtos Naturais, Departamento de Bioquímica, Centro de Ciências Biológicas, Universidade Federal de Pernambuco, Avenida Professor Moraes Rêgo s/n, Cidade Universitária, 50670-420 Recife, PE, Brazil

## Abstract

An increasing number of biological activities presented by medicinal plants has been investigated over the years, and they are used in the search for new substances with lower side effects. *Eugenia uniflora* L. and *Eugenia malaccensis* L. (Myrtaceae) have many folk uses in various countries. This current study was designed to quantify the polyphenols and flavonoids contents and evaluate the immunomodulatory, antioxidant, and cytotoxic potentials of fractions from *E. uniflora* L. and *E. malaccensis* L. It was observed that the polyphenol content was higher in ethyl acetate fractions. These fractions have high antioxidant potential. *E. malaccensis* L. seeds showed the largest DPPH radical scavenger capacity (EC_50_ = 22.62). The fractions of *E. malaccensis* L. leaves showed lower antioxidant capacity. The samples did not alter the profile of proinflammatory cytokines and nitric oxide release. The results indicate that species of the family Myrtaceae are rich in compounds with antioxidant capacity, which can help reduce the inflammatory response.

## 1. Introduction

The genus *Eugenia* is considered one of the largest belonging to family Myrtaceae (subfamily Myrtaceae) with about 500 species of trees and shrubs in tropical and subtropical America. Among these species, approximately 400 are distributed in Brazil, assuming an importance especially for providing extracts commonly used in traditional medicine with various therapeutic properties [1].

*Eugenia uniflora* L. has red fruits, which are used popularly as antihypertensive [2], as well as in the treatment of digestive disorders [3]. In some regions, are considered diuretic, hypotensive [4], anti-inflammatory, antidiarrheal [5], and antimicrobial [6].

*Eugenia malaccensis* L. (synonym: *Syzygium malaccensis*) is a species of flowering tree that is native to Malaysia, Indonesia, and southern Vietnam. The trees are beautiful, and although they do not originate from America, they are well adapted to tropical conditions, especially the northern and northeastern Brazil [7]. In many parts of the world, *E*. *malaccensis* has been used in folk medicine to treat various diseases and symptoms, including headaches, coughs, diabetes, inflammation, and hypertension [8].

Phytochemical studies with *Eugenia* species revealed the presence of flavonoids [9], tannins, terpenoids [10], and essential oils [11], whereas from the pharmacological point of view, other studies performed with crude extracts and compounds have proven anti-inflammatory, analgesic and antipyretic [12], antifungal [13], hypotensive [4], antihyperlipidemic [14], hypoglycemic [15], and antioxidant [16] activities.

The aim of this study was to evaluate the flavonoids and total phenolic content, as well as the antioxidant, hemolytic and immunostimulatory activities of semipurified fractions of leaves and seeds from *E*. *uniflora* L. and *E*. *malaccensis* L.

## 2. Methods

### 2.1. Collection of Plant Material and Preparation of Organic Extracts

Leaves and seeds from *E*. *uniflora* L. and *E*. *malaccensis* L. were obtained, after growth under natural conditions, from Bonito and Recife cities, in Brazil. The plant material is deposited in the herbarium Dárdano de Andrade-Lima (IPA), voucher specimen n° 83147. Powdered leaves and seeds from *E*. *uniflora* L. and *E*. *malaccensis* L. were subjected to the extraction of secondary metabolites, through technical of infusion, using methanol as organic solvent. The crude methanol extracts of leaves and seeds were used for fractionation, in order to separate its constituents. The materials were submitted to liquid-liquid partition with solvents ethyl acetate (EtOAc) (1 : 1) and n-butanol (n-BuOH) (1 : 1), at room temperature, resulting after evaporation of the solvents, and the semipurified samples: ethyl acetate fraction (EtOAc Fr.), butanol fraction (n-BuOH Fr.), and aqueous fraction (H_2_O Fr.). 

### 2.2. Dosage of Flavonoids and Polyphenols Content

The polyphenol content in extracts was quantified according to the Folin-Ciocalteau method [17], with a few modifications. Aliquots of test samples (0.05 mL) were mixed into test tubes with 1.0 mL of distilled water and 0.5 mL of Folin-Ciocalteau reagent (1 : 1 v/v). This mix was kept in the dark during 10 min, and 2.5 mL of sodium carbonate [Na_2_CO_3_] (20%) was added. The mixture was incubated for twenty minutes at room temperature, and the absorbance was measured at 735 nm using a spectrophotometer. Gallic acid was used as a standard. All tests were performed in triplicate. 

The determination of flavonoids content in the samples was performed according to the method described by [18], with a few modifications. Aliquots of sample (0.1 mL) in the concentration 10 mg/mL were mixed with 0.1 mL of aluminum trichloride [AlCl_3_] (20%) and one gout of acetic acid [C_2_H_4_O_2_]. Dilute the solution in methanol until the volume of 5 mL. After 40 min of incubation, the absorbance of the solutions was determined in a spectrophotometer at 415 nm. The mixture in absence of the aluminum trichloride was used as a blank. Quercetin in the concentration 0.5 mg/mL was used as a standard. All tests were performed in triplicate. 

### 2.3. DPPH Radical Scavenging Activity

DPPH-free radical scavenging activities of the extracts were determined according to the method of [19], with a few modifications. In plate with ninety-six wells was added 0.04 mL of diluted in methanol samples (1, 3, 10, 30, 100, 300, and 1000 *μ*g/mL) and 0.25 mL of the DPPH (2.22 mg/mL). After 30 min at room temperature and in the dark, absorbance was measured at 517 nm. For the blank was added 0.04 mL of solvent with 0.25 mL of DPPH solution. For the positive control was used 0.04 mL of quercetin in presence of the DPPH solution. 

Blood (5–10 mL) was obtained from healthy nonsmoking volunteers by venipuncture, after a written informed consent was obtained. Human erythrocytes from citrated blood were immediately isolated by centrifugation at 1500 rpm for 10 min at 4°C. After removal of plasma and buffy coat, the erythrocytes were washed three times with phosphate-buffered saline (PBS; pH 7.4) and then resuspended using the same buffer and a 1% erythrocyte suspension was prepared. The hemolytic activity of the crude extract was tested under in vitro conditions. Each tube received 1.1 mL of erythrocyte suspension and 0.4 mL of extract of various concentrations (50–500 *μ*g/mL) were added. The negative control was only solvent and the positive control received 0.4 mL of Quillaja sp. saponin (0.0025%). After 60-min incubation at room temperature, cells were centrifuged and the supernatant was used to measure the absorbance of the liberated hemoglobin at 540 nm. The average value was calculated from triplicate assays. The hemolytic activity was expressed in relation to Quillaja sp. saponin and calculated by the following formula:

(1) $$\begin{array}{c} \text{Hemolytic}\;\text{activity}\;\left(\%\right)=\left(\text{As}-\text{Ab}\right)\left(\text{Ac}-\text{Ab}\right)\times 100, \end{array}$$

where Ac was the absorbance of the control (blank, without extract), As was the absorbance in the presence of the extract, and Ac was the absorbance of saponin solution.

### 2.4. Animals

Male BALB/c mice (six-eight weeks old) were used in all experiments of immunomodulatory activity. They were obtained from the Oswaldo Cruz Foundation (FIOCRUZ, Rio de Janeiro, Brazil) and maintained at the animal facilities of the Aggeu Magalhães Research Center of the Oswaldo Cruz Foundation in Recife, Brazil. All mice were sacrificed and treated according to the Oswaldo Cruz Foundation guidelines for laboratory animals. This work was approved by the institutional ethics committee (protocol number 0266/2005).

### 2.5. Cytotoxicity Assay

To determine the nontoxic concentration of the samples, splenocytes from BALB/c mice (6 × 10^5^ cells/well) were cultured in ninety-six well plate in RPMI 1640 media supplemented with 10% fetal bovine serum and 50 *μ*g/mL of gentamycin. Each sample was evaluated in six concentrations (1, 5, 10, 25, 50, and 100 *μ*g/mL) in triplicates. Cells were incubated in the presence of  ^3^H-thymidine during 24 h at 37°C and 5% CO_2_. Two controls were made in this experiment: negative control (cells incubated with ^3^H-thymidine) and positive control (cells treated with saponin, a substance with known cytotoxic activity, and incubated with ^3^H-thymidine). After 24 h, the cultures were harvested, using a cell harvester to determine the ^3^H-thymidine incorporation in a beta radiation counter. The viability of the cells was determined by the ^3^H-thymidine incorporation of extracts-treated wells, and the cytotoxicity was calculated in relation to the 3H-thymidine incorporation of untreated cultures [21].

### 2.6. Analysis of Cell Viability by Annexin V-FITC and Propidium Iodide Staining

Splenocytes were incubated (10^6^ cells/mL) for 24 h with samples (5 *μ*g/mL). They were centrifuged at 4°C, 450 g for 10 min. After discarding the supernatant, 1 mL of PBS 1X was added to the precipitate, and it was then centrifuged at 4°C, 450 g for 10 min. The pellet was resuspended in the same buffer, and annexin V conjugated with fluorescein isothiocyanate (FITC) (1 : 500) and propidium iodide (PI, 20 *μ*g/mL; 10^6^ cells) were added, in the dark, to each cytometer tube. Afterwards, the samples were then analyzed (minimum of 10.000 events/tube) through flow cytometry (FACSCalibur-BD, San Jose, CA, USA) using the software CELLQuestProTM (BD Bioscience, San Jose, CA, USA) for acquisition and analysis of data. The results were represented in graphs by dot plot [21].

### 2.7. Determination of Cytokine Levels in Splenocytes Culture

The assay was made according to Pereira et al. [21]. Cells were cultured in plates with twenty-four wells at a density of 10^6^ cells/well in RPMI 1640 medium. IFN-*γ*, IL-2, and IL-6 cytokines were quantified in 24 h, 48 h, and 72 h and six days in the culture supernatants stimulated with samples at nontoxic concentrations established previously (25, 10, 5, and 1 *μ*g/mL). Negative (spontaneous secretion) and positive (concanavalin A) controls were included. The cytokine levels were quantified by sandwich enzyme-linked immunosorbant assay (ELISA), according to the manufacturer's suggested protocols.

### 2.8. Statistical Analysis

All tests were performed in triplicate. The concentration needed for 50% of inhibition (EC_50_) was estimated graphically by nonlinear regression analysis. Other data were analyzed through Mann-Whitney *U* test and were analyzed considering the value of *P* < 0.05 as statistically significant. The correlations between phytochemicals (polyphenols ad flavonoids) and antioxidant or hemolytic activities were calculated using the Pearson coefficient (*ρ*). 

## 3. Results and Discussion

An increasing number of biological activities presented by medicinal plants has been investigated over the years, and several experimental models are used in the search for new bioactive substances [22–24].

The phytochemical screening revealed the presence of flavonoids and tannins in all fractions analyzed, which is consistent with the research of Auricchio et al. [6]. The estimation of phenolic compounds revealed that all ethyl acetate fractions (EtOAc Fr.) exhibited the highest phenolic content, except the *E*. *malaccensis* L. leave EtOAc Fr. All *E*. *malaccensis* L. leave fractions showed the lowest amount of polyphenols (Table 1). The evaluation of the phenolic compounds uses the Folin-Ciocalteau reagent, which forms blue complexes in the presence of reducing agents [25]. The intensity of blue-colored complex is related to the presence of hydrogen donating groups in the phenolic compounds [26]. The highest phenolic content was found in the EtOAc Fr. and n-BuOH Fr., which is expected when performing the liquid-liquid fractionation with solvents of increasing polarity. The levels of flavonoids are higher in leaves of the two species. A major function of this group of compounds is to protect plants against ultraviolet, and as the leaves are more exposed than the seeds, the higher amounts of flavonoids in the leaves are justified (Table 1).

**Figure 1:**
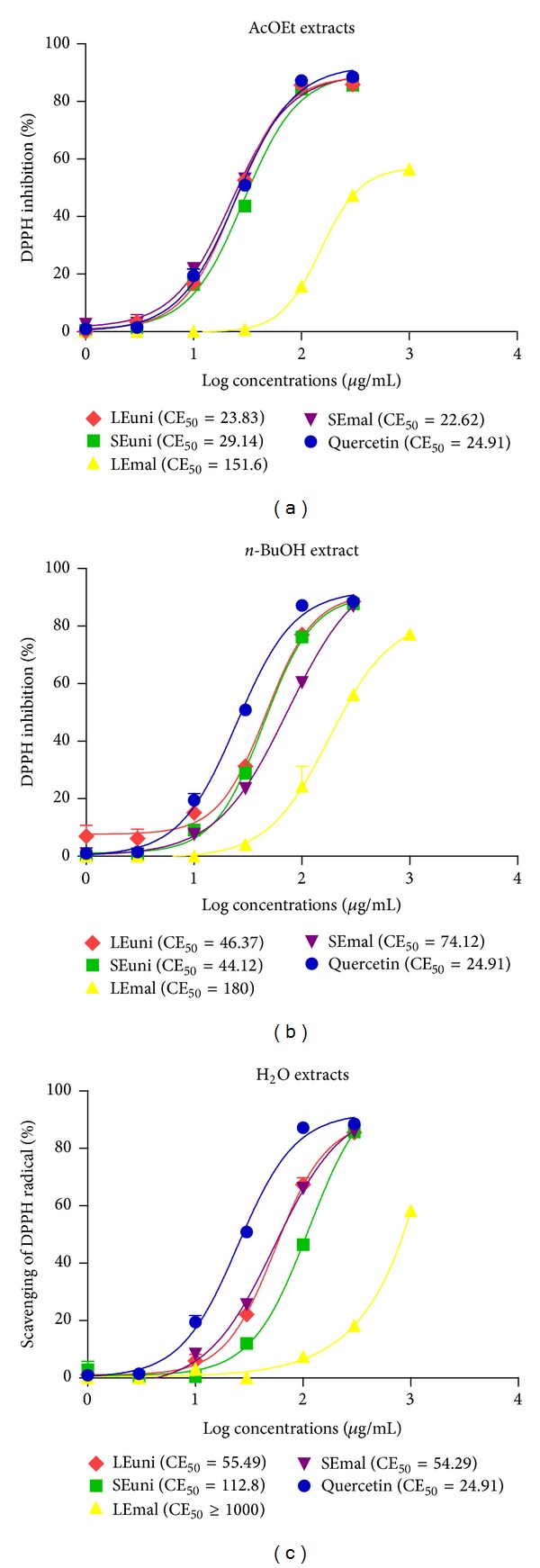
DPPH radical inhibition of different extracts from *E. uniflora* and *E. malaccensis*. (a) EtOAc Fr. (b) n-BuOH Fr. (c) H_2_O Fr. EC_50_ is the concentration needed for 50% of inhibition. The values are represented as mean ± SD (*n* = 3). Leaves *E. uniflora* (LEuni), seeds *E. uniflora* (SEuni), leaves *E. malaccensis* (LEmal), and seeds *E. malaccensis* (SEmal).

The total antioxidant activity of fractions was determined by the ability of antioxidants present in the samples to capture the stable DPPH radical (2,2-diphenyl-1-picril-hydrazyl). The method is based on the DPPH radical scavenger by antioxidants with conversion to its reduced form, consequently producing a decrease in absorbance. Thus, the methanol solution of DPPH, initially violet, turns yellow, and the degree of discoloration indicates the ability of the antioxidant-free radical scavenger [24]. According to the results obtained, we can say that the fractions of analyzed species have a high antioxidant potential. It is observed that the *E*. *malaccensis* L. SEtOAc Fr. has the highest capacity of DPPH radical scavenging, followed by *E*. *uniflora* L. LEtOAc Fr. (Table 1). EC_50_ values were lower than those of quercetin. The highest antioxidant capacity in n-BuOH Fr. was observed in *E. uniflora* L. leaves and seeds, while the H_2_O Fr. followed the same sequence of EtOAc Fr. (Figure 1). For both plants, the correlation of DPPH radical scavenging ability was stronger with polyphenols (*ρ* = −0.70 for *E. uniflora* and −0,737 for *E*. *malaccensis*) than flavanoids (*ρ* = −0.581 and −0.298 for *E*. *uniflora* and *E*. *malaccensis*). It is important to note that as the correlations with DPPH radical scavenging ability were determined using EC_50_, a negative *ρ* value (−1) is considered as perfect positive correlation. 

The hemolytic activity of fractions was performed to discard the possible cytotoxic mechanism and verify the safety of the phytocompound, making them appropriate for the preparation of natural drugs. Hemolytic tests were performed because the compounds that have biological effects may not be useful in pharmacological preparations, if they have hemolytic effect. In addition, these data can also reveal some information about the mechanism of cytotoxicity [27]. The results showed that fractions from *E*. *malaccensis* L. are more toxic than those from *E*. *uniflora* in the highest concentration used for this test. In addition, the EtOAc Fr. showed the greatest hemolytic effect when compared with n-BuOH Fr. and H_2_O Fr. (Table 1). Very strong correlations of hemolytic action of samples from *E*. *uniflora* and their polyphenols and flavonoids content (*ρ* = 0.961 and 0.853, resp.) were found, while for *E*. *malaccensis*, a moderate correlation was observed for both (*ρ* = 0.309 for polyphenols; *ρ* = 0.535 for flavonoids), suggesting the effect of other classes of compounds.

Furthermore, we assessed cytotoxicity of the partially purified extracts using a culture of spleen cells isolated from BALB/c mice. In the study of cytotoxicity, LEtOAc Fr. and Ln-BuOH Fr. from *E*. *malaccensis* L. and LEtOAc Fr. from *E*. *uniflora* L. showed toxic profile above 25 *μ*g/mL. LH_2_O Fr., Sn-BuOH Fr. (*E*. *malaccensis* L.), Ln-BuOH Fr., and Sn-BuOH Fr. (*E*. *uniflora* L.) presented toxicity above 50 *μ*g/mL. SEtOAc Fr. from *E*. *malaccensis* L. and *E*. *uniflora* L. was the most toxic sample (above 10 *μ*g/mL), whereas SH_2_O Fr. of the two species and LH_2_O Fr. of *E*. *uniflora* L. did not show a toxic profile at any concentrations tested (Table 2). 

**Figure 2:**
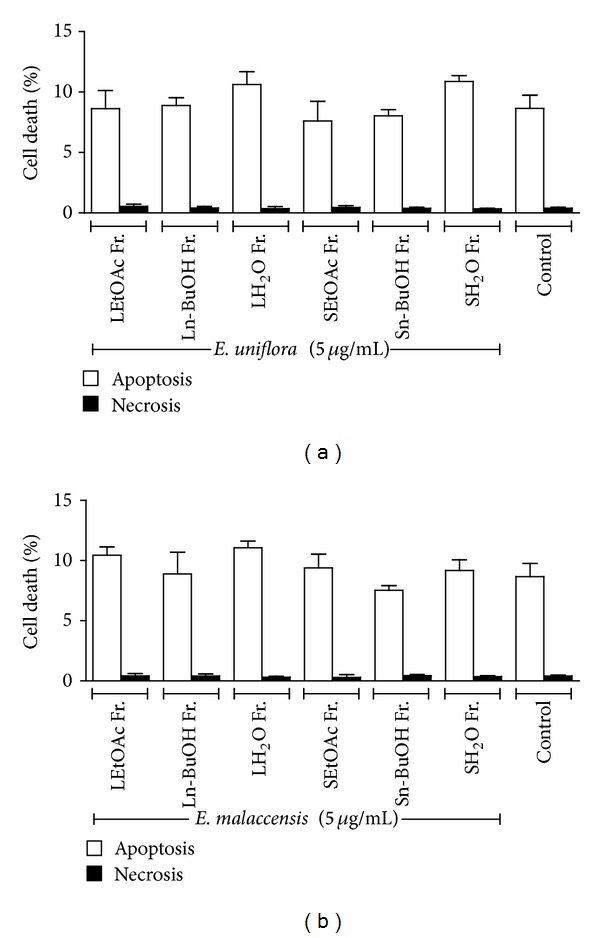
Percentage of apoptotic cells and necrotic cells treated with extracts from *E. uniflora* (a) and *E. malaccensis* (b). Values are given as mean ± SD (*n* = 4). Ethyl acetate fraction of leaves (LEtOAc Fr.), butanol fraction of leaves. (Ln-BuOH Fr.), aqueous fraction of leaves (LH_2_O Fr.), ethyl acetate fraction of seeds (SEtOAc Fr), butanol fraction of seeds (Sn-BuOH Fr.), and aqueous fraction of seeds (SH_2_O Fr.).

Several stimuli can induce cell death by one of the two existing routes: apoptotic or necrotic. Annexin V is responsible for differentiating apoptotic cells from viable cells, while PI is specific to recognize cells that have compromised plasma membrane integrity [28]. The cell viability assay using annexin V-FITC and PI showed that the extracts induced minor damage to cells, and after 24 h of assay, about 90% were still viable. We observed similarity between the pattern shown by untreated cells with those exposed to the extract, both with respect to apoptosis and necrosis. That is, the percentage of cells treated with the extract that in apoptosis or necrosis has behavior of cell death by apoptosis or necrosis was similar to control groups (Figure 2).

The results show that the profiles of cytokine (IFN-*γ*, IL-2, and IL-6) and nitric oxide released by primary splenocytes were not altered by any concentration of the tested fraction in relation to unstimulated cultures (date not shown). The low T_H_1 cytokines production observed in this study may indicate that the two Myrtaceae species did not induce a proinflammatory response. These results are corroborated by other studies that demonstrated that polyphenols, especially from fruits, exhibited inhibitory effects on the expression and activity of enzymes involved in the generation of inflammatory mediators, such as NO [29, 30]; they are associated with the highest inhibition of T_H_1 cytokines [31] and promote T_H_2 response [32].

In conclusion, this work indicates that *E*. *malaccensis* L. and *E*. *uniflora* L. are rich in compounds with antioxidant capacity, which can help to reduce the inflammatory response, through different signaling pathways which can be activated in vivo. The isolation and characterization of new substances from these plants, with lower side effects, may help in the treatment of diverse pathologies.

## Figures and Tables

**Table 1:** Flavonoid and total phenolic content and hemolytic activity of extracts from *E. uniflora* and *E. malaccensis*.

Extract	Total phenolic content (mgGAE/g)	Flavonoid content (mgQE/g)	Hemolysis (%)
***E. uniflora***			
LEtOAc Fr.	2756 ± 15.5	84.3 ± 3.4	72.73 ± 0.96
Ln-BuOH Fr.	2492 ± 58.1	8.6 ± 0.9	1.9 ± 0.72
LH_2_O Fr.	2445 ± 15.5	2.1 ± 0.07	0
SEtOAc Fr.	2831 ± 23.0	31.2 ± 1.1	72.23 ± 0.68
Sn-BuOH Fr.	2365 ± 38.3	5.6 ± 0.4	2.1 ± 0.74
SH_2_O Fr.	2351 ± 12.3	2.9 ± 0.4	0
***E. malaccensis***			
LEtOAc Fr.	1641 ± 31.9	40.7 ± 1.5	100
Ln-BuOH Fr.	565 ± 32.7	20.7 ± 6.6	100
LH_2_O Fr.	264 ± 9.5	2.0 ± 0.4	0
SEtOAc Fr.	2920 ± 24.4	5.8 ± 0.3	100
Sn-BuOH Fr.	2341 ± 36.5	2.9 ± 0.9	100
SH_2_O Fr.	2256 ± 37.3	0	3.39 ± 0.82

Legend: (LEtOAc Fr.): ethyl acetate fraction of leaves, (Ln-BuOH Fr.): butanol fraction of leaves, (LH_2_O Fr.): aqueous fraction of leaves, (SEtOAc Fr.): ethyl acetate fraction of seeds, (Sn-BuOH Fr.): butanol fraction of seeds, and (SH_2_O Fr.): aqueous fraction of seeds. Values are means ± SD (*n* = 3).

**Table 2:** Cytotoxicity assay using splenocytes from BALB/c mice treated with extracts of *E. uniflora* and *E. malaccensis*.

Extract	Concentrations (*μ*g/mL)					
	1	5	10	25	50	100
*E. uniflora *						
LEtOAc Fr.	−	−	−	−	+	+
Ln-BuOH Fr.	−	−	−	−	−	+
LH_2_O Fr.	−	−	−	−	−	−
SEtOAc Fr.	−	−	−	+	+	+
Sn-BuOH Fr.	−	−	−	−	−	+
SH_2_O Fr.	−	−	−	−	−	−
*E. malaccensis *						
LEtOAc Fr.	−	−	−	−	+	+
Ln-BuOH Fr.	−	−	−	−	+	+
LH_2_O Fr.	−	−	−	−	−	+
SEtOAc Fr.	−	−	−	+	+	+
Sn-BuOH Fr.	−	−	−	−	−	+
SH_2_O Fr.	−	−	−	−	−	−
Saponin**	+	+	+	+	+	+

Legend: *Nontoxic concentrations were determined by ^3^H-thymidine incorporation. Percentages below 30% compared to the immune cells not treated (control) are considered nontoxic (−). (LEtOAc Fr.): Ethyl acetate fraction of leaves, (Ln-BuOH Fr.): butanol fraction of leaves, (LH_2_O Fr.): aqueous fraction of leaves, (SEtOAc Fr.): ethyl acetate fraction of seeds, (Sn-BuOH Fr.): butanol fraction of seeds, and (SH_2_O Fr.): aqueous fraction of seeds.
